# Editor's Reply

**DOI:** 10.1371/journal.pmed.0020423

**Published:** 2005-12-27

**Authors:** Emma Veitch

**Affiliations:** **1**PLoS Clinical TrialsCambridgeUnited Kingdom

Erick Turner appropriately points out the high levels of rigor applied during regulatory authorities' review of clinical trial data [1]. However, the statement beginning “However, it is difficult to have confidence in data released by sponsors…” [2] was not intended to highlight the release of review documents by the Food and Drug Administration (FDA), but rather the publication of summary clinical trial data on sponsors' own Web sites, which does seem to lack an integral peer-review mechanism. I support efforts to make Drugs@FDA more systematic and comprehensive, an initiative which can sit comfortably alongside peer-reviewed journal publication.
